# Mixed Modeling of Meta-Analysis P-Values (MixMAP) Suggests Multiple Novel Gene Loci for Low Density Lipoprotein Cholesterol

**DOI:** 10.1371/journal.pone.0054812

**Published:** 2013-02-06

**Authors:** Andrea S. Foulkes, Gregory J. Matthews, Ujjwal Das, Jane F. Ferguson, Rongheng Lin, Muredach P. Reilly

**Affiliations:** 1 Division of Biostatistics, School of Public Health and Health Sciences at the University of Massachusetts, Amherst, Massachusetts, United States of America; 2 Cardiovascular Institute, Perelman School of Medicine at the University of Pennsylvania, Philadelphia, Pennsylvania, United States of America; Medical University Hamburg, University Heart Center, Germany

## Abstract

Informing missing heritability for complex disease will likely require leveraging information across multiple SNPs within a gene region simultaneously to characterize gene and locus-level contributions to disease phenotypes. To this aim, we introduce a novel strategy, termed Mixed modeling of Meta-Analysis P-values (MixMAP), that draws on a principled statistical modeling framework and the vast array of summary data now available from genetic association studies, to test formally for locus level association. The primary inputs to this approach are: (a) single SNP level p-values for tests of association; and (b) the mapping of SNPs to genomic regions. The output of MixMAP is comprised of locus level estimates and tests of association. In application of MixMAP to summary data from the Global Lipids Gene Consortium, we suggest twelve new loci (PKN, FN1, UGT1A1, PPARG, DMDGH, PPARD, CDK6, VPS13B, GAD2, GAB2, APOH and NPC1) for low-density lipoprotein cholesterol (LDL-C), a causal risk factor for cardiovascular disease and we also demonstrate the potential utility of MixMAP in small data settings. Overall, MixMAP offers novel and complementary information as compared to SNP-based analysis approaches and is straightforward to implement with existing open-source statistical software tools.

## Introduction

Serum lipid levels are established determinants of cardiovascular disease morbidity with well-described heritability. Indeed, meta-analysis of data arising from recent genome-wide association studies has identified common genetic variants in at least 95 loci associated with low-density lipoprotein cholesterol (LDL-C), high-density lipoprotein cholesterol (HDL-C), triglycerides (TG) and total cholesterol (TC) [1]. In practice, selection of statistically relevant genes or loci, subsequently referred to collectively as loci, is often based on simple linear regression of single-nucleotide polymorphisms (SNPs); that is, loci within which there is at least one SNP that reaches genome-wide significance, defined according to a Bonferroni level correction for multiple testing, are regarded as significantly associated with the trait under study. While this approach is valid, we conjecture that substantial, complementary knowledge about association can be acquired by considering available information on all SNPs within a locus simultaneously in characterizing association.

In order to address this, we apply a mixed effects modeling paradigm that uses SNP-level meta-analysis p-values to arrive at formal analytic characterization of underlying locus-level effects on complex disease phenotypes. Through application of principled, well-vetted statistical concepts for valid and reliable inference, this approach, termed **Mix**ed modeling of **M**eta-**A**nalysis **P**-values (**MixMAP**), draws strength from available information across all SNPs within a pre-defined region without requiring first-stage data reduction. That is, it is not necessary to eliminate redundancy among SNPs in high linkage disequilibrium (LD) within a locus. By incorporating all available information about SNPs within a locus, MixMAP results in increased sensitivity for identifying loci with multiple SNPs of moderate significance, as evidenced in the applications described below.

Applications of hierarchical linear and generalized linear models for analysis of data arising from genetic association studies have been described previously. Specifically, use of mixed effects models for family studies is common to account for correlation arising from familial level clustering where a random effect for family is included in the model (see for example [2], [3]). In the context of population-based investigations of unrelated individuals, applications of mixed models are described with random effects for SNPs, genes and sets of genes (see for example [4]–[8]). A primary conceptual distinction between MixMAP and alternative approaches is that MixMAP uses summary level 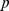-values as the dependent variable in the hierarchical model rather than raw data, allowing investigators to leverage existing, publicly-accessible data resources. MixMAP also provides locus level tests and these are not limited by an increasing number of SNPs within a gene as described in [7]. Technically most similar to MixMAP, the approach of Wang *et al.* [8] involves fitting a hierarchical model to summary level data, specifically 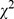-statistics from single-SNP tests of association; however, in addition to differences in model specification, hypothesis testing within the Wang *et al.* framework is based on fixed effects parameters (representing pathway effects) while MixMAP involves generating prediction intervals for latent (locus-level) effects.

The purpose of this manuscript is to describe the MixMAP algorithm and to illustrate its application under a range of conditions. Application of MixMAP to two independent datasets of SNP associations with LDL-C, a causal risk factor for atherosclerotic cardiovascular disease (CVD), reveals that MixMAP offers novel and complementary information as compared to traditional analysis approaches. Notably, as an analytic tool that selects loci with multiple moderate to strong signals, and one that uses single SNP-level summary data (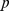-values), MixMAP is intended to serve as an additional analysis framework that complements, rather than replaces, single-SNP based investigations. This is further supported by simulation studies suggesting MixMAP has a higher true positive rate than single-SNP based analysis in the context of more moderate gene level signals. MixMAP is straightforward to implement with the open-source MixMAP package in R (freely-available for download at http://cran.r-project.org/ and http://people.umass.edu/foulkes/software.html.).

## Results

### Summary approach

This section reports the results of applying MixMAP to data arising from two independent sources: the Global Lipids Gene Consortium (GLGC) and the Penn Coronary Artery Calcification (PennCAC). These revealed that application of MixMAP: (1) supports published LDL-C loci; (2) suggests novel LD-CL genes and (3) complements a single-SNP testing approach, as described in detail below. Additionally, the results of simulation studies designed to characterize the performance of MixMAP under a range of conditions, also described below, support the concept that MixMAP serves as a complementary analysis strategy. To begin, we briefly outline the MixMAP algorithm and the available GLGC and PennCAC data. Further details are provided in the Material and Methods.

#### Summary of the MixMAP algorithm

MixMAP is a statistical framework that uses the results of single SNP-level analysis to test formally for locus-level association. The MixMAP approach is designed to identify loci involving multiple SNPs each with moderate effects on the trait that may not be detected by single SNP analysis. Loci that are detected by a single SNP approach may also be detected by MixMAP if multiple SNPs have modest association across the locus. The primary inputs to the MixMAP algorithm are: (a) p-values corresponding to single SNP tests of association with a trait (e.g. LDL-C); (b) a mapping of SNPs to genomic regions. Additional SNP and gene-level covariate information, such as gene size, number of SNPs per Kb, number of recombinant hotspots per Kb and average linkage disequilibrium can be incorporated.

The MixMAP algorithm is summarized as follows, with additional detail provided below: [**Step 1:**] Fit a mixed effects model to inverse normally transformed (ranked) 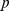-values, with random locus-specific intercepts; [**Step 2:**] Predict random locus-specific effects using empirical Bayes estimation; [**Step 3:**] Calculate corresponding prediction intervals using a Bonferroni corrected threshold; and [**Step 4:**] Report a locus as statistically meaningful if the upper limit of the prediction interval corresponding to the locus effect is less than 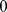. In the present manuscript, we fit the mixed model with random gene-level effects and provide a post-hoc characterization of loci (genes or groups of genes) based on the genes that emerge through application of MixMAP. As more comprehensive information on the relationship between genes within loci becomes available, the MixMAP approach is flexible in that an alternative loci-level annotation can be used as input to the algorithm.

#### Summary of the GLGC and PennCAC data

The current applied investigation focuses on loci for LDL-C, an important causal factor for CVD. Data arising from two independent data-sets are considered: (1) reported and publicly available meta-analysis SNP level 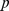-values for association with LDL-C derived from multiple independent association of approximately 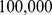 individuals in the Global Lipids Gene Consortium (GLGC) study (http://www.broadinstitute.org/mpg/pubs/lipids2010/); and (2) SNP level 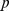-values for association with LDL-C derived from analysis of ITMAT-Broad-CARe (IBC) 50K SNP array data in European ancestry individuals within the Penn Coronary Artery Calcification (PennCAC) sample [9]–[11], a relatively small study (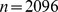 Caucasians) that is considered underpowered by itself to identify the genetic determinants of a complex disease phenotype. Additional details on this cohort are provided in Supporting Information S1.

A total of 31827 SNPs in 2960 genes that are common to both GLGC and PennCAC are used in the current investigation. As an illustrative example, we chose to focus on the set of SNPs included on the IBC array which was specifically designed to provide SNP coverage in putative candidate CVD genes as well as emerging loci at the time of design [12]. This facilitates (a) focus on a defined set of SNP within candidate loci and (b) direct comparison of findings across the two datasets that were examined. Representing the largest published lipids meta-analysis, GLGC significant SNPs are treated as the “gold-standard” to which application of MixMAP to PennCAC is compared. Further validation of the findings resulting from applying MixMAP to GLGC is not practical presently given the comprehensive inclusion of all prior genome wide association studies in GLGC, although this should be feasible in the near future with published and available larger datasets from on-going projects such as the GLGC Metabochip project [13]. Instead, however, we use published literature on human data, animal/mouse models and cell biology to support our suggested novel discoveries.

### Application of MixMAP in GLGC supports published LDL loci

Here we describe our interpretation of MixMAP findings in assigning genes to already established or novel loci in GLGC analysis. Briefly, we assigned MixMAP identified genes to an established GLGC locus (marked by SNP in GLGC Table 1 [1]) if LD 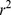 values were 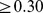 for one or more SNPs in a MixMAP identified gene and a GLGC top SNP. Additionally, MixMAP identified genes that were in close physical proximity (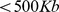) to a GLGC top SNP but did not have strong LD (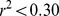) or were within a wider region with multiple candidate genes (e.g. HLA) were also assigned to the GLGC established LDL-C locus. The SNP Annotation and Proxy Search (SNAP) [14] web-based tool (http://www.broadinstitute.org/mpg/snap/) (for SNPs 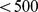
Kb apart) and the Genome-wide LInkage DisEquilibrium Repository and Search engine (GLIDERS) [15] (for SNPs 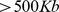 apart) were used to determine pairwise LD between interrogated SNPs. In SNAP, we used the SNP dataset for the 1000 Genomes Pilot 1 [or HapMAP 3 (release 2) build 36 for SNPs not available in 1000 Genomes] and population panel “CEU” while in GLIDERS HapMAP 3 (release 2) build 36 was used.

**Table 1:** Contingency table representing measures of predictive accuracy for simulations studies.

		Actual		
		Informative	Non-informative	Total
Predicted	Informative	TP	FP	(TP + FP)
	Non-informative	FN	TN	
	Total:	*m* = (TP + FN)	(FP + TN)	*N*

Simulation studies report: TPR (sensitivity)  =  TP/(TP+FN); FPR (1-specificity)  =  FP/(FP+TN); and FDR  =  FP/(TP+FP).

**Figure 1:**
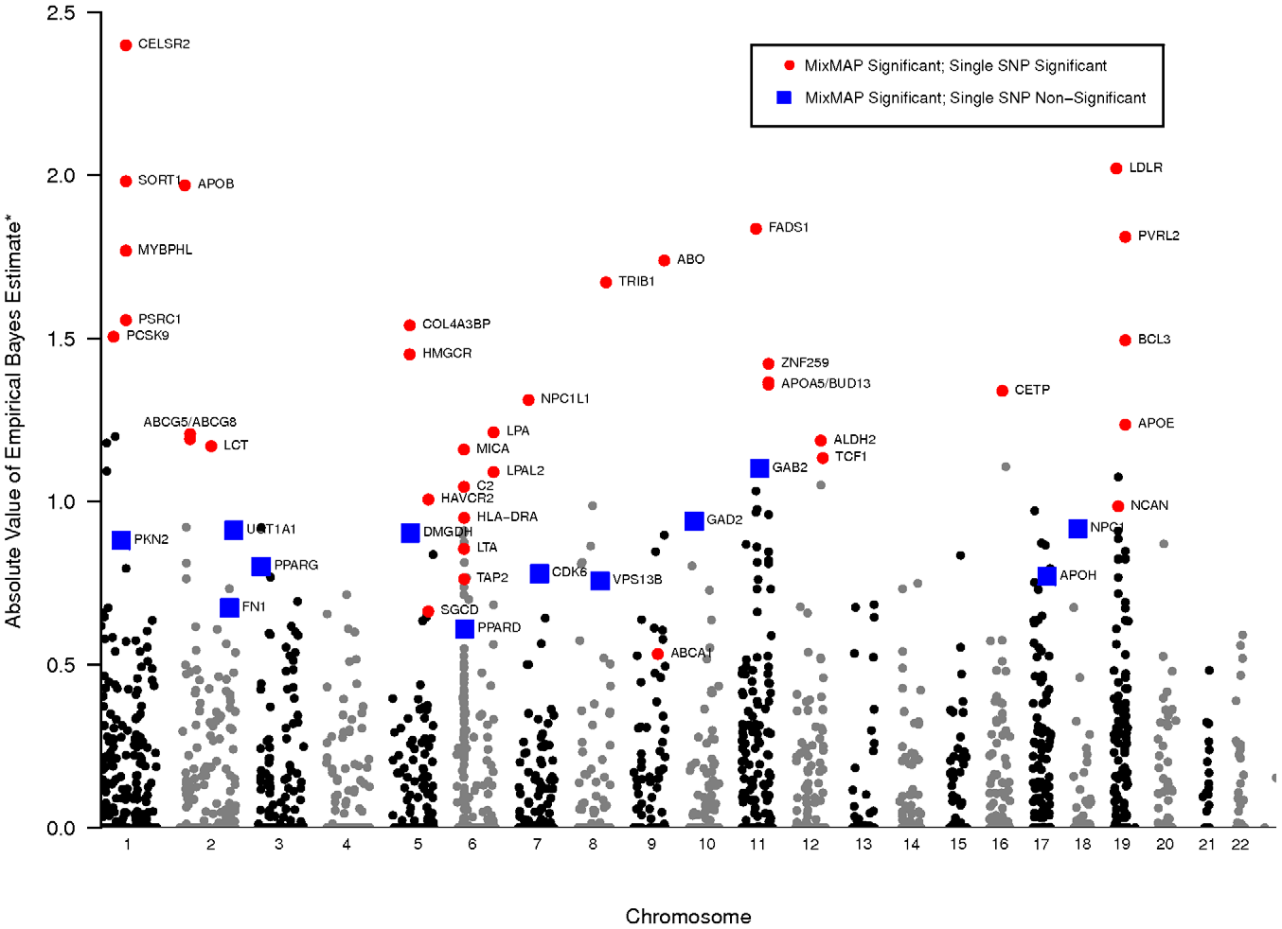
MixMAP gene-level effects for GLGC data. Points in this Manhattan style plot represent genes with their approximate location on the x-axis and their corresponding effect estimates on the y-axis for the 2960 genes interrogated in Global Lipids Genetic Consortium (GLGC) summary data [1]. Genes that are detected by both MixMAP and single SNP analysis are represented by red circles. Unique MixMAP findings for genes that lack single SNP association signals are highlighted with blue rectangles. Grey and black dots are genes not detected by either the single SNP or MixMAP approaches. After a conservative multiple testing adjustment, MixMAP identifies 12 loci in GLGC that are not identified by single SNP analysis. *The absolute value of EB estimates are reported with positive values set to 0. Negative inverse normal transformed *p*-values that are large in absolute value correspond to small *p*-values on the original scale. Corresponding prediction variances and interval limits are provided in Table S1.

Analysis of 31827 SNPs in 2960 genes in GLGC data identified 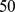 genes in 26 loci based on a single SNP signal threshold of 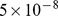 (Table 2). MixMAP detects 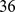 genes within 21 of these 26 loci based on a Bonferroni corrected gene-level threshold of 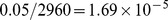 (Table 2; Figure 1; Table S1). Thus, of 26 LDL-C loci detected by the single SNP approach in GLGC GWAS data, 21 were also detected by MixMAP while 5 were not detected. A comparison of MixMAP detected and not detected GLGC genes/loci is of some interest to illustrate the strengths and weaknesses of the approach. First, coverage of the genes at loci detected by single SNP analysis but not detected by MixMAP is generally lower than those detected by both approaches (5 vs. 13 median number of SNPs respectively). Second, genes detected by a single SNP signal but not MixMAP tend to have a higher median SNP p-value (median  =  0.006 for genes with greater than 2 SNPs) compared to genes detected by both approaches (median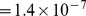) suggesting that the distribution of p-values of SNPs within genes detected by both approaches is shifted downwards compared to that of genes detected by a single SNP only signal (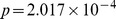 for two-sided Wilcoxon rank sum test of difference in medians). Importantly, from a biological perspective, almost all clinically important LDL-C genes/loci were detected (e.g., LDLR, APOB, APOE, HMGCR, PCSK9, LPA, SORT1, ABCG5/8, TRIB1, ABCA1, APOA5-A4-C3-A1 and CETP) while genes at loci not detected (LDLRAP1, ANGPTL3, HFE, HPR, TOP1) tended to be less well characterized functionally and clinically.

**Table 2:** MixMAP results in GLGC for IBC array loci with evidence for single SNP LDL-C association.

		Position						Single SNP p-values		
Locus‡	Chr	Start	Stop	Gene Name‡	Single SNP^*1^	MixMAP^*2^	# of SNPs	Min	Median	Max
LDLRAP9	1	25568422	25749764	RHCE	+	-	4	3.10E-10	2.07E-05	1.98E-003
	1	25762009	25767440	LDLRAP1	+^a^	-	5	6.38E-05	3.17E-04	3.59E-002
PCSK9	1	55267951	55304223	PCSK9	+	+	34	1.93E-28	1.22E-05	0.8714
ANGPTL3	1	62704220	62822181	DOCK7	+	-	3	2.16E-17	3.17E-17	8.62E-017
	1	62827868	62845701	ANGPTL3	+	-	6	1.43E-17	0.18	0.973
SORT1	1	109745416	109745601	PSMA5	+	-	2	1.56E-12	–	8.78E-011
	1	109590236	109623689	CELSR2	+	+	23	9.70E−171	2.31E-29	0.7796
	1	109622442	109630036	PSRC1	+	+	7	4.93E-164	1.44E-08	0.3477
	1	109633806	109650249	MYBPHL	+	+	10	7.89E-28	1.68E-12	0.07608
	1	109652649	109742656	SORT1	+	+	28	1.63E-23	1.14E-12	0.8676
APOB	2	21052397	21165196	APOB	+	+	49	4.48E-114	7.51E-18	0.9401
ABCG5/8	2	43921795	43958429	ABCG8	+	+	21	1.73E-47	0.001336	0.9203
	2	43893343	43924284	ABCG5	-	+	25	8.14E-08	0.07115	0.4809
RAB3GAP1^†^	2	136262314	136307216	LCT	-	+	11	1.13E-05	5.36E-05	0.6756
HMGCR	5	74667257	74693036	HMGCR	+	+	11	5.12E-45	5.70E-13	0.7414
		74711473	74793312	COL4A3BP	+	+	9	2.90E-35	2.07E-12	0.4902
TIMD4^†^	5	155681482	156120506	SGCD^c^	-	+	66	3.38E-07	0.113065	0.8402
	5	156445860	156469146	HAVCR2^c^	-	+	11	0.003134	0.009268	0.5286
HFE	6	26196869	26204727	HFE	+	-	8	6.07-10	5.92E-03	6.64E-001
HLA	6	32512043	32535726	HLA-DRA	+	+	13	7.28E-13	0.01082	0.7028
	6	31469689	31498389	MICA	-	+	20	2.60E-06	0.011105	0.6733
	6	31644203	31652541	LTA	-	+	18	0.0002275	0.01893	0.9282
	6	32000620	32025519	C2	-	+	14	3.97E-05	0.041215	0.4409
	6	32899566	32917826	TAP2	-	+	25	4.81E-07	0.116	0.8873
LPA	6	160810340	160838285	LPAL2	-	+	9	2.38E-06	0.0008496	0.6485
	6	160873025	161011583	LPA	+	+	29	1.36E-15	1.36E-05	0.9738
NPC1L1	7	44519763	44551551	NPC1L1	+	+	14	4.93E-11	7.35E-05	0.9729
TRIB1	8	126506812	126573908	TRIB1	+	+	49	2.83E-29	7.98E-10	0.9683
ABO	9	135197039	135229220	SURF1	+	-	5	1.57E-12	1.60E-02	1.49E-001
	9	135121293	135145180	ABO	+	+	15	4.60E-21	0.0003062	0.01966
ABCA1	9	106585724	107556417	ABCA1	+^b^	+	121	1.12E-07	0.2582	0.979
FADS1-2-3	11	61305135	61361791	FADS1	+	+	9	1.75E-21	8.41E-21	0.0006352
	11	61353788	61389758	FADS2	+	-	6	2.12E-20	5.75E-04	7.88E-001
	11	61398293	61420267	FADS3	+	-	7	8.80E-10	3.72E-04	6.20E-001
APOA5-A4-C3-A1	11	116024949	116145447	BUD13	+	+	6	4.21E-99	4.91E-06	0.05234
	11	116152068	116168917	ZNF259	+	+	9	1.47E-26	4.01E-09	0.8141
	11	116157417	116170289	APOA5	+	+	6	2.32E-16	7.16E-09	0.04245
	11	116172547	116202948	APOA4	+	-	8	1.97E-08	0.069	0.732
	11	116212611	116233487	APOA1	+	-	12	1.18E-09	4.12E-03	0.839
	11	116230513	116233840	APOC3	-	-	2	7.93E-03	–	0.665
BRAP^†^	12	110368991	110368991	SH2B3	+	-	1	1.73E-09	–	–
	12	110714419	110731337	ALDH2	+	+	7	5.42E-09	5.92E-06	0.4789
	12	110971201	110971201	C12orf30	+	-	1	6.89E-09	–	–
HNF1A^†^	12	119890027	119923981	TCF1	+	+	13	3.61E-15	0.03823	0.437
CETP	16	55548996	55576893	CETP	+	+	44	1.64E-12	0.003372	0.942
HPR	16	70640155	70671503	HPR	+	-	6	1.75E-22	1.35E-05	2.84E-001
LDLR	19	10663792	10663792	ILF3^c^	+	-	1	2.01E-14	–	–
	19	11024562	11024562	SMARCA4	+	-	1	1.74E-25	–	–
	19	11063306	11103658	LDLR	+	+	28	4.28E-117	6.98E-10	0.5643
CILP2	19	19185443	19329924	NCAN	+	+	12	1.42E-19	0.06043	0.5713
	19	19324032	19366087	KIAA0892	+	-	5	1.78E-15	5.68E-04	3.58E-001
	19	19515117	19524850	CILP2	+	-	5	5.99E-21	0.506	0.631
	19	19526643	19584215	PBX4	+	-	7	2.52E-18	6.89E-02	4.81E-001
	19	19580840	19619190	EDG4	+	-	7	1.02E-17	0.065	0.051
APOE-C1-C2	19	49929652	49944944	BCL3	+	+	6	4.21E-99	4.91E-06	0.05234
	19	50021054	50021054	BCAM	+	-	1	6.18E-63	–	–
	19	50043777	50074877	PVRL2	+	+	16	5.11E-67	1.05E-08	0.6158
	19	50081014	50119488	APOE	+	+	8	3.76E-110	2.67E-07	0.9906
	19	50100676	50100676	TOMM40	+	-	1	3.76E-110	–	–
	19	50139001	50145079	APOC4	+	-	5	1.08E-72	6.08E-03	0.458
	19	50139018	50149020	APOC2	+	-	7	2.56E-10	0.074	0.652
TOP1^†^	20	39225477	39230879	PLCG1	+	-	2	5.99E-15	–	0.764

For the 31827 SNPs in 2960 genes interrogated in Global Lipids Genetic Consortium (GLGC) summary data [1], MixMAP detects 36 genes in 21 of 26 loci detected by single SNP analysis in GLGC. Coverage of genes detected by both approaches is higher, while the median p-value is generally lower, than that of genes detected by single SNP analysis alone. ‡Genes were assigned to a locus identified in the GLGC [1] if SNPs in that gene were within 500 Kb of or had linkage disequilibrium (LD) 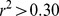 with the top SNP at a GLGC genome wide significant locus. ^†^Genes designated at genome wide significant locus in GLGC were not directly interrogated in IBC array data; 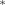 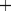 indicates corresponding gene detected and – indicates corresponding gene not detected; 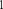
genome wide significant threshold (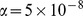); 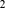
Bonferroni correction based on the number of genes (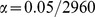); 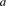
Gene is significant based on Single SNP approach in [1] (Table 1) but significant SNP is not included in IBC array under study; 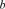
This gene is significant based on single SNP approach for TC and HDL in GLGC [1] (Table 1) and conditionally associated with LDL (Table 6 Supp); 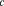
Limited LD (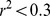) detected; however, assigned to same locus due to physical proximity (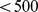
Kb) and/or multiple candidate genes in the region. These may represent signal for LDL-C independent of the established GLGC locus.

### Application of MixMAP in GLGC suggests novel LDL genes

Twelve (12) additional loci are supported by MixMAP that are not detected using the single SNP signal threshold in the GLGC data (Table 3 and Figure 1). While the minimum 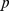-values within index genes at these loci are more moderate than those detected by the single SNP threshold, in that they do not reach genome-wide significance, the overall distribution of 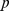-values is lower than expected under no association (one-sided Wilcoxon signed rank test that the median of the within gene 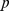-values is greater 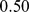, against the alternative that it is less than 0.50, 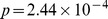.) The median 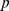-value of SNPs within these genes ranges from 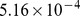 for a gene with 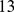 SNPs to 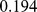 for a gene with relatively high coverage of 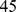 SNPs. Several of the genes in this group (e.g. PPARG, PPARD and NPC1) are strongly implicated in lipid and lipoprotein metabolism in human and animal model systems, as indicated and referenced in the final column of Table 4 (See Supporting Information S2 for more detail).

**Table 3:** MixMAP in GLGC identifies IBC array loci that lack single SNP evidence for LDL-C association.

		Position						Single SNP p-values			Supporting
Locus	Chr	Start	Stop	Gene Name	Single SNP^*1^	MixMAP^*2^	# of SNPs	Min	Median	Max	Evidence^§^
PKN2	1	88918822	89073711	PKN2	-	+	21	0.003906	0.06777	0.6081	None
FN1	2	215938326	216066837	FN1	-	+	44	1.56E-05	0.1254	0.9993	^†^B [49], [50]
UGT1A1	2	234255355	234346384	UGT1A1	-	+	20	3.53E-06	0.070225	0.9471	A [18]–[20]; B [21]
PPARG	3	12309416	12450557	PPARG	-	+	49	1.35E-05	0.1021	0.9917	?A,B,C[22]–[25]
DMDGH	5	78331610	78402461	DMGDH	-	+	19	0.002283	0.08308	0.6427	^‡^A [51]
PPARD	6	35419966	35503982	PPARD	-	+	45	0.002272	0.1943	0.8976	A [31]–[34]; B [52]
CDK6	7	92074765	92304241	CDK6	-	+	43	0.0006457	0.1083	0.8932	None
VPS13B	8	100143528	100936497	VPS13B	-	+	30	0.0003567	0.101375	0.861	^**^A [42]
GAD2	10	26552050	26631994	GAD2	-	+	22	0.006998	0.06269	0.3543	None
GAB2	11	77604417	77802894	GAB2	-	+	13	1.65E-05	0.0005164	0.9401	None
APOH	17	61629102	61657177	APOH	-	+	25	2.10E-05	0.1556	0.9846	A [26]–[29]
NPC1	18	19366772	19415132	NPC1	-	+	16	0.014	0.04201	0.3832	A,B,C [35]–[38]

For the 31827 SNPs in 2960 genes interrogated in Global Lipids Genetic Consortium (GLGC) summary data [1], 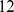 novel loci are detected by MixMAP alone. The median p-values for these genes tend to be lower than expected by chance. ^†^No association with plasma lipids but mouse models support role in atherosclerosis; ^‡^No association with plasma lipids but cause of inborn error of choline metabolism; ^**^No association with plasma lipids but implicated in Cohen syndrome in which truncal obesity is a feature; ?PPARG is a prominent gene for insulin resistance, type-2 diabetes mellitus, lipodystrophy and obesity; some data link PPARG to lipoprotein abnormalities; 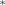 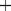 indicates corresponding gene detected and – indicates corresponding gene not detected; 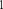
genome wide significant threshold (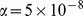); 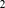
Bonferroni correction based on the number of genes (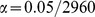); 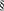
Based on published literature (see corresponding citations): A: human data; B: mouse/animal data; and C: cell biology.

**Table 4:** Top MixMAP and single SNP evidence for IBC array locus association with LDL-C in PennCAC.

		Position			PennCAC Results			Top GLGC Findings		Supporting
Locus	Chr	Start	Stop	Gene Name	Single SNP^*1^	MixMAP^*2^	# of SNPs	SNP	p-value	Evidence^§^
ELA2A	1	15653807	15669301	ELA2A	+	-	8	rs10927787	0.048	None
RGS7	1	239219450	239268557	RGS7	+	-	38	rs628208	0.008	None
MFSD7	4	671940	671940	MFSD7	+	-	1	rs9991613	0.671	None
ESR1	6	152167137	152467893	ESR1	+	-	149	rs9341052	1.83E-04	A [53]–[56]
APOA5-A4-C3-A1	11	116024949	116145447	BUD13	+	-	16	rs6589565	5.37E-16	A [1]
	11	116157417	116170289	APOA5	+	-	6	rs2075290	2.32E-16	A [1]
YY1	14	99795191	99809982	YY1	+	-	2	rs4905941	0.232	C [57]–[59]
FEM1B	15	66348570	66371610	FEM1B	+	-	7	rs16951723	0.352	None
SORT1	1	109590236	109623689	CELSR2	-	+	23	rs629301	9.70E-171	A [1]
IL1R2	2	101970201	102010893	IL1R2	-	+	37	rs2236927	0.086	A [39]; B [40]; C [41]
TNIP3	4	122257475	122313818	TNIP3	-	+	14	rs17051298	0.058	None
FGF2	4	123975987	124033758	FGF2	-	+	25	rs308406	0.039	None
LPA	6	160873025	161011583	LPA	-	+	28	rs10455872	1.36E-15	A [1]
GRM3	7	86106844	86327561	GRM3	-	+	32	rs10245069	0.058	None
VPS13B	8	100143528	100936497	VPS13B	-	+	30	rs7841688	3.57E-04	A^†^ [42]

For the 31585 SNPs in 2944 genes interrogated in PennCAC, MixMAP identifies 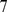 loci in PennCAC that are not identified by single SNP analysis. Of these gene/loci, SNPs in 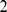 reach genome wide significance in GLGC (SORT1 and LPA) and are also MixMAP significant in GLGC; 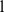 is MixMAP significant but single SNP non-significant in GLGC (VPS13B), and 2 have animal model data supporting modulation of lipid metabolism (VPS13B and IL1R2). ^†^No association with plasma lipids but implicated in Cohen syndrome in which truncal obesity is a feature;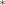 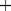 indicates corresponding gene detected and – indicates corresponding gene not detected; 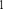
IBC array threshold (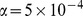); 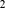
Bonferroni correction based on the number of genes (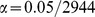); 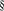
Based on published literature (see corresponding citations): A: human data; B: mouse/animal data; and C: cell biology.

### Application of MixMAP in small sample setting complements single SNP testing approach

Here we apply MixMAP to a small study (PennCAC) that generated IBC array SNP data. In terms of independent support, we treat published GLGC data as the gold standard to which PennCAC is compared. This analysis includes 31585 (of the original 31827 SNPs) in 2944 (of 2960) genes that remain after filtering out those with MAF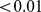 and HWE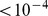. As might be anticipated for this small sample, no SNPs meet genome-wide significance (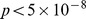) or even IBC array-wide significance (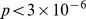; an estimated threshold for independent SNP tests based on simulations [16]) for association with LDL-C using the single SNP signal approach. However, 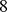 genes in 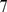 loci contain SNPs that are significant at the 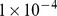 level, a previously applied threshold for suggestive stage 1 evidence of association in IBC studies [16]. Notably, in PennCAC, none of these 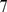 loci coincide with significant MixMAP locus tests at the Bonferroni corrected threshold of 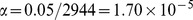; however, 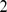 interrogated genes (BUD13 and APOA5) in 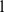 of these 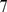 loci (APOA5-A4-C3-A1) do have single SNP signals for LDL-C at the Bonferroni corrected level in the GLGC study data [1]. The same 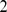 genes within this locus had significant MixMAP findings in GLGC data.

**Figure 2:**
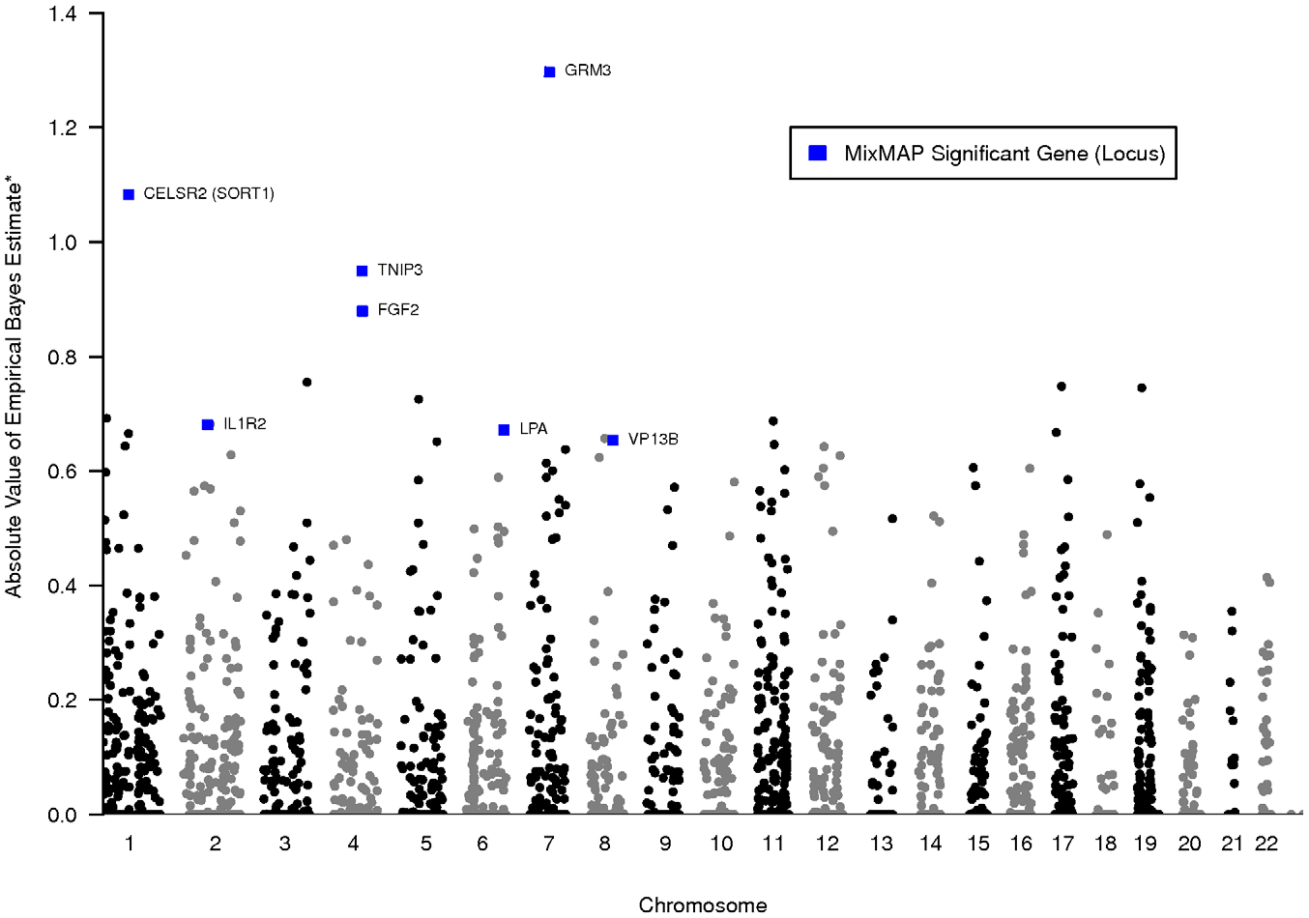
MixMAP gene-level effects for PennCAC data. Points in this Manhattan style plot represent genes with their approximate location on the x-axis and their corresponding effect estimates on the y-axis for the 2944 genes interrogated in PennCAC. No individual SNPs met genome wide or array wide significance in these data. After a conservative multiple testing adjustment, MixMAP identifies 7 loci in PennCAC that are not identified by single SNP analysis. These MixMap findings are highlighted with blue rectangles. All other genes are represented by grey and black circles. *The absolute value of EB estimates are reported with positive values set to 0. Negative inverse normal transformed *p*-values that are large in absolute value correspond to small *p*-values on the original scale. Corresponding prediction variances and interval limits are provided in Table S2.

In PennCAC, an additional 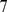 genes in 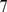 distinct loci are supported by MixMAP based on the Bonferroni corrected threshold of 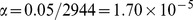 (Table 4 and Figure 2). None of these loci have single SNP tests that reach even the suggestive threshold of 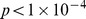 in PennCAC; however, 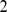 of these 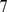 loci reach genome wide significance based on single SNP signals in GLGC (SORT1 and LPA). The same 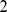 loci, as well as VPS13B had significant MixMAP findings in GLGC data. Furthermore, 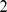 loci (IL1R2, VPS13B) have some support for modulation of lipids in animal models, as indicated in the final column of Table 3. Thus, in this PennCAC data-set, an illustrative example for small sample settings, MixMAP may add value to single SNP based testing in identifying loci for LDL-C and other complex traits.

### Simulation studies support concept of MixMAP as a complementary strategy to single SNP analysis

In order to evaluate the performance of MixMAP relative to single SNP analysis, we investigate the ability to detect informative loci and the likelihood of false findings as functions of gene level effect size (measured by the shift parameter in a two-component Gaussian mixture distribution), the number of informative genes (measured by the number of genes with random effects arising from a non-zero mean normal prior) and coverage (measured by the proportion of observed SNPs included in the analysis). Details of the simulation approach are described in Materials and Methods below. Simulation results are reported in terms of: the true positive rate (**TPR**), defined as the proportion of true signal genes that are correctly identified; the false discovery rate (**FDR**), given by the proportion of selected genes that are not associated with the trait; and the false positive rate (**FPR**), defined as the proportion of truly uninformative genes that are incorrectly selected as significant. Explicit definitions of TPR, FDR and FPR are given in Table 1. In all simulation scenarios, the results of applying MixMAP as well as the single SNP approach are presented to illustrate the potential gains associated with using MixMAP as a complementary strategy.

**Figure 3:**
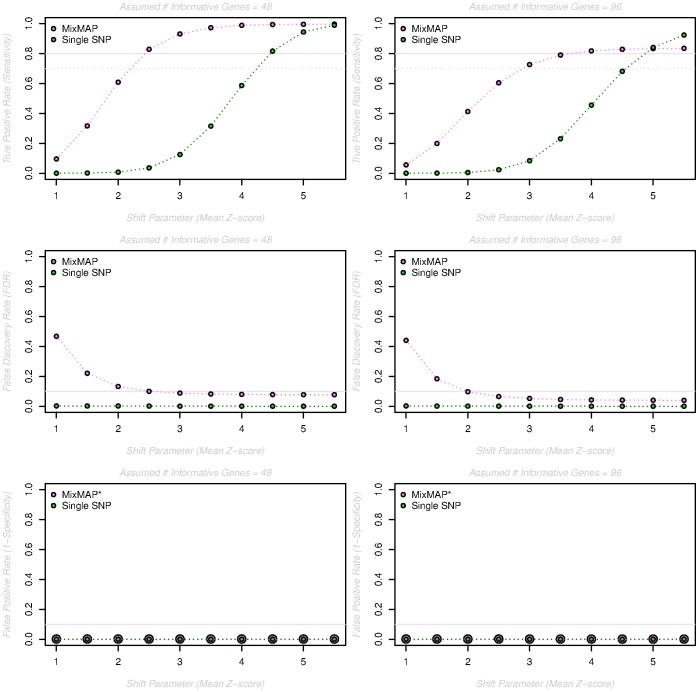
Simulation results for a range of shift parameters and number of informative genes. The true positive rate (TPR, row 1), false discovery rate (FDR, row 2) and false positive rate (FPR, row 3) are reported (y-axis) for shift parameters ranging from 1 to 5.5 (x-axis), when the “true” (under simulation) number of informative genes is equal to 48 (left hand column) or 96 (right hand column). “Informative” genes are assumed to have effects that arise from a normal distribution with mean equal to the shift parameter while all remaining gene-level effects (2960 total) arise from a standard normal distribution. All estimates are based on 500 simulations per condition. *Dots are enlarged to visualize overlapping symbols.

**Figure 4:**
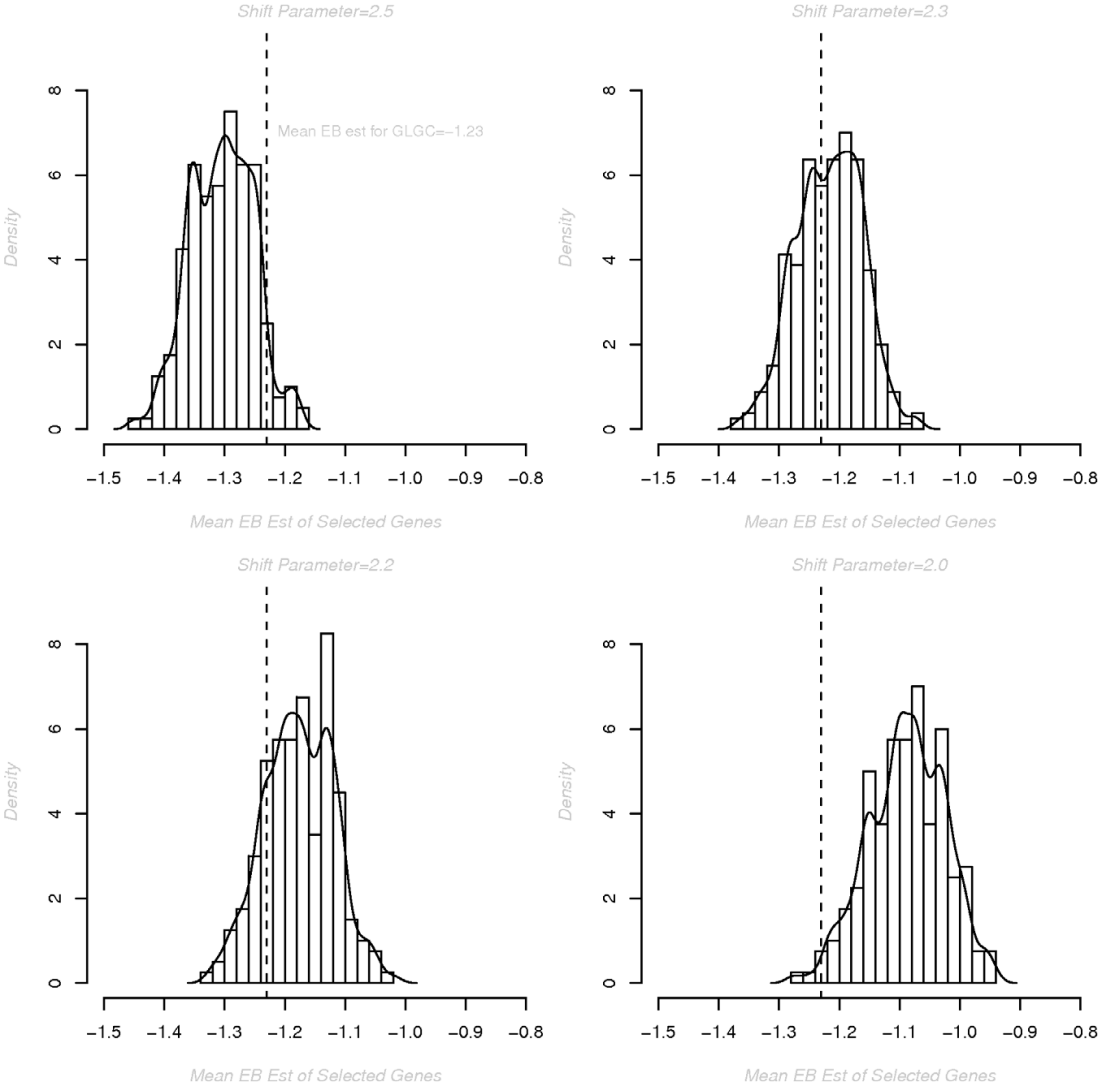
Density plots for average EB estimates of selected genes for a range of shift parameters. Density plots represent the distribution of the average Empirical Bayes (EB) estimate across MixMAP selected genes when the effects of 48 genes are assumed to arise from a normal distribution with mean equal to the indicated shift parameter while all remaining gene-level effects (2960−48 = 2912) arise from a standard normal distribution. Each plot is based on 2000 simulations. The mean of the EB estimates for the GLGC MixMAP selected genes was −1.23 (indicated by the dotted vertical line in the top left panel). This is most consistent with an underlying shift parameter of approximately 2.3.

Figures 3 illustrates the estimated TPR (Sensitivity), FDR and FPR (1-Specificity) for shift parameters ranging from 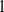 to 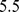, where estimates are based on 500 simulations per condition. The left panel of this figure reports results when the true number of informative gene is 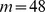, the number detected in the GLGC data, while the right panel considers twice as many (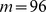) informative genes. The range of 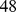 to 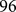 informative genes was selected because it is generally consistent with the number of significantly associated loci in prior reports of complex disease traits. Notably for the GLGC data, the mean empirical Bayes (EB) (shrinkage) estimate for the 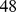 detected genes was 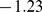. This is consistent with a shift parameter of approximately 2.3, as reported in Figure 4; however, a broader range of shift parameters is presented in the simulations studies for illustration and generalizability.

As expected, the TPR increases with increasing shift parameter values, with MixMAP consistently higher than the single-SNP based approach for more moderate shift parameters (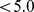). For both the single-SNP and MixMAP approaches the rate of increase in the TPR (as a function of the shift parameter) is slower when the number of informative genes is 96 versus 48, though the difference is more pronounced in the context of MixMAP. This result is consistent with underlying statistical theory, as the estimated variance parameter for the single normal prior distribution of the random effects will be larger as more genes arise from an alternative distribution with non-zero mean. As a result, the corresponding gene-level prediction intervals will be wider and it will be more difficult to detect informative genes. This result suggests that as the number of truly informative genes increases, the added contribution of MixMAP over single SNP analysis is smaller; however, with as many as 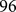 informative genes, MixMAP is detecting a greater percentage of truly informative genes than the single SNP approach for shift parameters less than 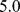. While the FDR is consistently low for the single-SNP based approach regardless of the shift parameter, the FDR for MixMAP is relatively high (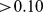) for shift parameters less than 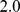. Notably, the average number of detected genes (across the 500 simulations) that are truly uninformative (FPs) is relatively constant across shift parameter values, ranging between 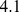 to 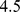 with a median of 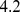. At the same time, the FDR tends to decrease because the total number of detected genes (TPs+FPs) is increasing as the shift parameter gets larger. Finally, the FPR is consistently small for both the single-SNP based and MixMAP approaches across all shift parameter values with averages (assuming 48 truly informative genes) of 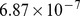 and 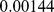, respectively.

**Figure 5:**
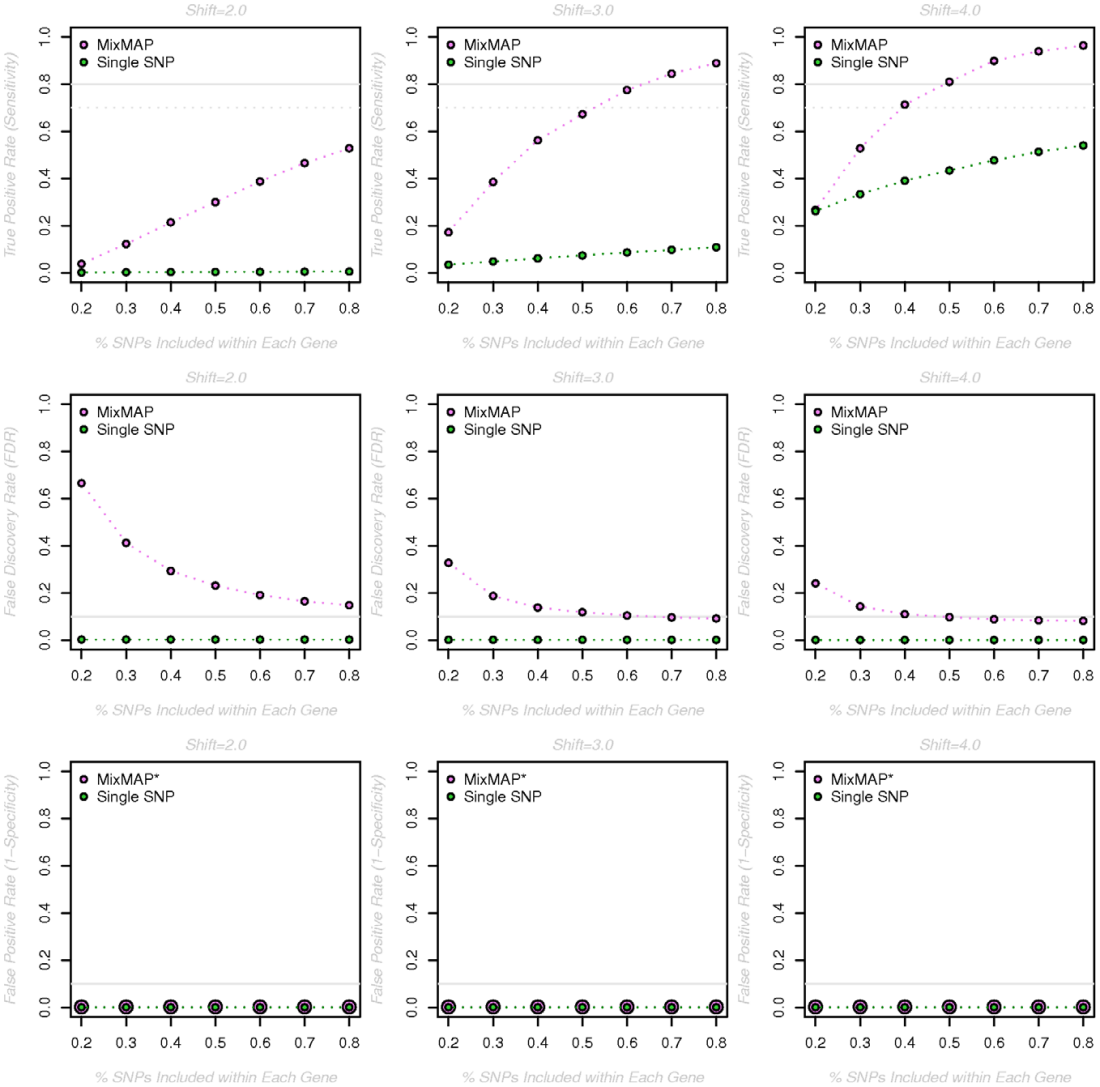
Simulation results for a range of shift parameters and proportions of SNPs included within a gene. The true positive rate (TPR, row 1), false discovery rate (FDR, row 2) and false positive rate (FPR, row 3) are reported (y-axis) for the percentage of SNPs included within each gene ranging from 0.2 to 0.8 (x-axis), when the “true” (under simulation) shift parameter is equal to 2.0 (left most column), 3.0 (middle column) or 4.0 (right most column). In these simulations 48 genes are considered “informative”. That is, the effects of these genes are assumed to arise from a normal distribution with mean equal to the shift parameter while all remaining gene-level effects (2960−48 = 2912) arise from a standard normal distribution. All estimates are based on 500 simulations per condition. *Dots are enlarged to visualize overlapping symbols.

Figure 5 reports the estimated TPR, FDR and FPR when only a subset of the observed SNPs within each gene is included in the analysis. The proportion of included SNPs (given on the x-axis) ranges from 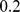 to 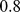 and each column in this figure represents a different value of the shift parameter (2.0, 3.0 or 4.0 for columns 1, 2 and 3 respectively). As expected for all values of the shift parameter, the TPR increases dramatically for MixMAP as the proportion of SNPs within a gene approaches 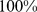. This follows from the dependency of the prediction interval for a gene level random effect on the number of SNPs within the corresponding gene. Further the MixMAP FDR is relatively high (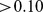) for low proportions (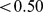) of included SNPs within each gene, and this is more pronounced for small shift parameters. Notably, while the FDR is high, the average number of detected genes (across the 500 simulations) that are truly uninformative (FPs) is relatively constant across the proportion of SNPs included, ranging between 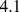 to 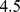 with a median of 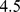 for shift parameter of 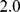. Finally, the FPR is consistently small for both the MixMAP and single-SNP approaches, with estimated values similar to those reported above with complete SNP data. These results suggests that the contribution of MixMAP will be more pronounced when genes are adequately covered, and application of MixMAP in the context of substantial missing information on SNPs can result in a higher proportion of false discoveries (relative to the total number of discoveries), though the absolute number of false discoveries remains constant.

## Discussion

Despite advances in the genomics of complex traits, only a portion of heritability for common human diseases has been elucidated. To date, most common variant discovery approaches have relied on tests of disease association using one SNP at a time. Methods that leverage existing datasets and exploit information across multiple SNPs within a gene region are likely to yield additional information regarding the locus association with traits. We developed a novel strategy, MixMAP, that relies on well-vetted statistical principles and draws from the vast array of summary data now available from genetic association studies, to test formally for locus-level association. The primary inputs required for this approach are single SNP level p-values for tests of trait association and mapping of SNPs to locus regions while the output is locus level estimates and tests of association. Application of MixMAP to SNP summary data for a pre-defined set of genes within the GLGC meta-analysis of association with LDL-C suggest that MixMAP can provide substantial value in discovery that is complementary to single SNP testing approaches in identifying novel loci for LDL-C. In addition, MixMAP analysis of PennCAC IBC array and LDL-C data support its application in combination with traditional SNP testing to enhance the power of discovery in small dataset settings. Thus, MixMAP provides a novel strategy, based on established statistical principles, for exploiting existing and emerging genomic data to provide advances in our understanding of complex human diseases.

Over the past decade sequencing of the human genome, definition of common SNP variation in human population and advances in genotyping technology have provided the possibility to discover common genetic contributions to complex traits in human. Indeed, very large scale applications of genome SNP scans in humans combined with rigorous statistical correction for multiple testing has led to an explosion of novel validated genomic discoveries for human diseases with exciting progress in functional genomics as well as promise for novel therapeutics and disease prediction. Despite this the majority of heritability for most complex traits remains to be discovered. Current statistical approaches for testing single SNP associations with disease are designed to protect against excess false positives but may be excessively conservative. Further, single SNP approaches to analysis do not draw strength from information gained by assessing simultaneously trends of association across a locus. These observations suggest that false negatives are a significant feature of existing association analysis and that additional genomic discovery should be possible in existing data if appropriate statistical methodologies are applied. Indeed, recent research suggests that common variants with individual level effects that are too small to be considered statistically significant using stringent significance thresholds account for a substantial proportion of this missing heritability for complex traits [17]. However, differentiating the true signals within the vast amount of SNP data with moderate p-values remains an unsolved problem.

We chose to analyze genetic contributors to LDL-C for several reasons. First, LDL-C is an important complex trait that is causal for a substantial portion of CVD death and morbidity in our society. Second, LDL-C has a well described heritability and large rigorously performed meta-analyses have been performed and summary data are publicly available (GLGC). Third, although many loci for LDL-C have been identified through association studies at 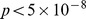, only a modest portion of its heritability (approximately 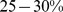 of genetic variability [1]) has been defined. Fourth, the basic biology of plasma lipids and LDL-C has been extensively studied in animal models and cell systems providing some additional mechanistic reference for any novel discoveries we might make. We hypothesized that we would identify novel loci for LDL-C, beyond the existing single SNP-based discoveries, through application of MixMAP in the large GLGC meta-analysis summary data. As an informative example, we chose to focus on the set of SNPs for CVD candidate genes included on the ITMAT-Broad-CARe (IBC) SNP array which was designed to provide dense SNP coverage in putative candidate CVD genes as well as some coverage of emerging loci at the time of its design [12]. This approach allowed us to focus on a defined set of SNPs within candidate loci and to perform direct comparison of findings for this subset of SNPs within the GLGC data-set to those in the smaller PennCAC sample application.

In GLGC data, MixMAP confirmed association for over 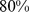 of the loci identified through single SNP testing of the 31827 SNPs in 2960 genes examined. Failure to detect more of the loci established by single SNP testing should not be surprising because MixMAP loses information for extremely low individual SNP p-values and is not designed for finding association when SNP coverage of a gene region is poor, as is the case for some loci that reached significance in the GLGC. Further, from a biological perspective, almost all clinically important LDL-C genes/loci were detected by MixMAP (e.g., LDLR, APOB, APOE, HMGCR, PCSK9, LPA, SORT1, ABCG5/8, TRIB1, ABCA1, APOA5-A4-C3-A1 and CETP). MixMAP, however, did provide evidence for 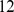 new loci (corresponding to 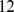 interrogated genes) for LDL-C in GLGC data that did not reach genome wide significance in single SNP testing. This may be an under estimate because we applied conservative criteria for our selection of novel loci (greater than 500kb from known GLGC locus, pairwise 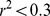 with top SNP at GLGC established LDL-C locus, and outside of region with multiple candidate genes). For example, interrogated SNPs in C2, which MixMAP identifies as a gene associated with LDL-C, have 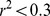 with the top GLGC SNP at the HLA locus in Teslovich et al. [1].

A more detailed description of the 12 genes/loci detected by MixMAP alone is provided in Supplementary Materials. For many of these 12 loci (FN1, UGT1A1, PPARG, GAB2 and APOH) the GLGC single SNP test p-value provided suggestive evidence of association (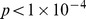) and published data in mice and human support specific biological processes and plausible mechanisms of association with LDL-C for some of the index genes (UGT1A1, PPARG and APOH) at these loci [18]–[29]. Notably, a recent meta-analysis of IBC array data for plasma lipids across 66,240 individuals also supports an association of APOH with LDL-C and suggests that UGT1A1 is a locus for total cholesterol levels [30]. For some MixMAP significant loci with suggestive GLGC single SNP tests, there is no or limited published biology or mechanism for association with LDL-C (e.g. FN1 and GAB2). On the other hand, some loci that are significant by MixMAP have quite modest statistical support in GLGC single SNP analysis, but have strong published data supporting mechanisms by which genes at the locus may modulate LDL-C (e.g NPC1 and PPARD) [31]–[38]. Finally, a few MixMAP loci have neither suggestive single SNP support from GLGC nor reported biological plausibility for gene-lipid associations (e.g. PKN2 and CDK) and such loci require further focus and validation. Overall, these data support the utility of MixMAP, when used in combination with traditional single SNP testing, in discovery of true loci for LDL-C and other complex traits particularly.

A specific challenge in the genomics of complex traits is identifying loci for such a trait when power is low due to limited availability of human data. We chose to illustrate this issue in a small sample (PennCAC, n = 2096) using LDL-C as an example in part because the large GLGC dataset for LDL-C provides an external reference for any MixMAP findings. In PennCAC, no individual SNPs meet criteria for association with LDL-C using the conservative genome-wide Bonferroni correction (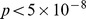) or the less conservative IBC array-wide Bonferroni correction (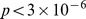). At a less stringent, suggestive single SNP criteria (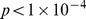), 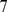 loci (represented by 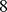 genes) are identified. At one of these loci, 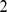 interrogated genes (APOA5 and BUD13) contain SNPs with genome-wide significant signals in the independent GLGC dataset. As expected at this less conservative threshold, however, most SNPs lack supporting signals in GLGC data and lack supporting biology for genes at the locus (Table 3) suggesting that several may be false positives. Using MixMAP 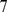 genes, representing 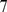 independent loci, were suggested for LDL-C. Of these, 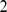 (SORT1 and LPA) have genome-wide significant signals in the independent GLGC data and one (VPS13B) had a significant MixMAP signal in GLGC data. Furthermore, 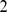 loci (IL1R2 and VP13B) have some support for modulation of lipids in animal models [39]–[42]. Overall, these PennCAC LDL-C analyses suggest that application of MixMAP in small sample settings may provide complementary value to single SNP tests and other strategies to maximize genetic inference in settings where sample size is limited. Although in these small sample settings false positives will remain a challenge, MixMAP should enhance findings for prioritization and further follow-up.

We recognize that independent replication of findings is essential for complete validation of novel findings in genetic studies. Because the GLGC data represent the largest published lipids GWAS meta-analysis to date, we believe a comprehensive replication for LDL-C beyond these GLGC data is not possible at the current time. However, we will pursue this for lipid genes/loci in additional GLGC data when these data become available (e.g., Metabochip project data expected 2013 [12]). We also acknowledge that for common SNP variation, a single gene often can not specifically be assigned to the disease-associated variant. Further, simple proximity to a variant and even incorporation of expression QTL knowledge are not always correct in selecting causal genes. This problem can lead to incorrect assumptions of causal genes and raise concerns for validity of gene-based inference. However, this limitation is not unique to our illustration of MixMAP and is common to current gene and pathway analyses leveraging common SNP datasets (e.g. [43]). The challenge can be addressed in part by leveraging the maximum amount of linkage disequilibrium, eQTL, fine mapping and biological data when assigning genes to the associated SNPs. In the present investigation, we use gene as the cluster to which SNPs belong, though MixMAP is not limited by this specification. Importantly, the user can employ alternative and newly evolved classifications, as the primary input to the MixMAP algorithm.

The results of the simulation study further support the application of MixMAP as a complementary strategy to single-SNP based testing, particularly in the context of moderate gene level effects and adequate SNP coverage. Our on-going research is exploring calibrating the variance coefficient in the prediction interval, as an alternative to using 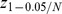, to obtain desired control of the FDR in specific well-defined settings. Because a first stage ranking of 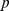-values is applied prior to inverse normally transforming the data for model fitting, the implications of using p-values from a single cohort study (PennCAC) versus a meta-analysis (GLGC) are limited to the varying degrees of precision in each setting. That is, the full range of the quantitative data, and specifically the fact that p-values from a meta-analysis tend to be substantially smaller than those from a single cohort study, is not being incorporated into the analysis presented herein. We expect additional knowledge can be gained through a mixture modeling extension of MixMAP that can accommodate the quantitative nature of the summary data, and this is currently under investigation. The present investigation is based on common variants, and while incorporating the results of rare variant analysis poses an additional challenge as these variants tend to be grouped *a priori* for analysis, such an extension would also likely be informative.

Further extensions of MixMAP would also allow application to gene set and/or pathway-based analysis of association data. Specifically, through inclusion of multiple nested random effects, the MixMAP framework could be applied using both locus level and pathway information simultaneously. Through fully parametric modeling, this may offer advantages over gene set enrichment analysis, which similarly involves a first stage rank ordering [44]. This extension of MixMAP would be notably distinct from the hierarchical modeling approach of [8] that similarly includes random gene specific effects, but separately models each gene set and focuses testing on fixed intercepts representing pathway effects rather than latent variables. Additional future work includes a specific evaluation of the influence of linkage disequilibrium, minor allele frequencies, gene size and numbers of recombinant hotspots as potential covariates in the models, as well as comprehensive evaluation of the complex statistical power considerations across a range of applications and conditions, including candidate gene studies, GWAS, pathway analysis and partial or whole-exome sequencing studies. Additional characterization of MixMAP may facilitate applications to summary findings from Metabochip and exome sequencing, as well in interrogation of gene sets and pathways utilizing such data. In conclusion, the approach we have described is intended to complement single SNP analysis and should provide a useful tool to potentiate existing summary data and reveal important novel loci, pathways and causal factors for complex diseases at little additional cost.

## Materials and Methods

As a consequence of the LD structure within genetic loci, we expect SNP level 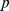-values, corresponding to single tests of association, to be potentially more similar within a gene than across genes, regardless of the level of association. Thus, a common statistical modeling framework for correlated data, the mixed effects model [45]–[47], is a natural analytic framework to consider for this setting. The application of MixMAP presented herein is at a gene level, and thus the term “gene” is used throughout this section; however, we note that as additional, locus-level annotations become available, these can replace or enhance the gene level classifications.

We begin by transforming the SNP level analysis or meta-analysis 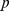-values, which will serve as the outcomes in our model, to normal variates in order to meet model assumptions. This is achieved by: (a) applying a simple rank transformation, to ensure uniformity over the interval from 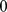 to 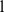; and (b) applying an inverse normal (probit) transformation to normalize the data. For (a), the rank of the 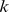
th SNP is set equal to 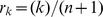 where 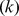 is the ordered ranking across all SNPs and 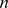 is the total number of SNPs under study. For (b), we let 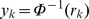 where 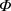 is the cumulative density function of a standard normally distributed random variable.

The first step of MixMAP is to fit a mixed effects model to appropriately transformed 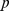-values with gene-specific random intercept terms and fixed effects for any relevant covariates. Formally this model is given by:
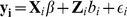
(1)where 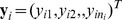, 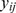 is the transformed 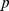-value for the 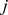
th SNP within gene 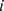, 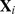 is a matrix with 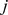
th row equal to a 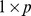 vector of SNP or gene level covariates, 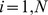 and 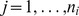, where 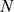 is the total number of genes and 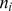 is the number of SNPs in gene 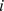. Further, we let 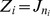 be an 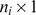 vector of 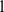
's, 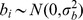 is the random effect of gene 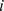, 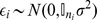 and 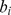 is independent of 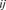. Finally, 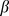 is the corresponding vector of fixed effects. In the example presented in this manuscript, the 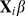 term additionally reduces to an overall intercept, but we retain this for generalizability. In this model, 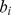 represents a latent (unobservable) effect of gene 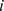 on the corresponding transformed 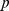-values. Notably, as a result of the transformation described above, small 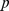-values correspond to large negative values of 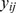. Thus, values of 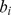 that are less than 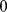 would indicate a gene level effect.

Based on this model formulation, the best linear unbiased predictor of the random effect for gene 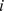 is given by:
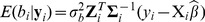
(2)where 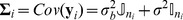 where 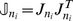. The empirical Bayes estimate of 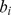, denoted 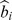, is calculated by replacing 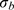 and 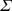 with corresponding REML estimates. A measure of dispersion for this quantity in the intercept only model is given by:
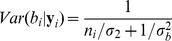
(3)and returned by the lmer() function in the R lme4 package (http://cran.r-project.org/web/packages/lme4/index.html). This is related in expectation to 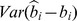 as described in [48], Chapter 7. Notably, the 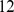 novel gene and locus findings reported in this manuscript were not sensitive to choice of prediction variance; however, if 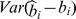 were applied in place of the measure returned by lmer(), then three genes (namely HAVCR2, HLA-DRA and LPAL2 in the TIMD4, HLA and LPA loci, respectively) would not be MixMAP significant.

In general, we are interested in testing the null hypothesis of no association between a given gene and the trait. To this aim, we construct a one-sided prediction interval for the true gene-level effect, given by 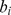 for gene 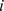, with the upper limit defined as:
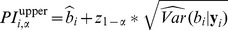
(4)where 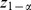 is the (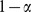)-quantile from a standard normal distribution, 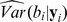 is equal to 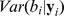 evaluated at RML estimates of 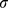 and 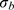, and 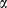 is a pre-defined significance threshold. A Bonferroni level threshold given by 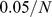 where 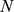 is the number of genes under study is suggested and applied in this manuscript. If the upper limit of the gene-level prediction interval is less than 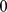, then we conclude that the corresponding locus is significantly associated with the trait.


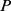
-values corresponding to tests of association with LDL-C were generated as follows, and according to the approach described in [1]: (1) Regress LDL on age, age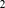, gender and the first 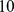 principal components derived using all available SNPs; (2) Calculate the residuals from this model fit; (3) Fit a separate simple linear regression for each SNP (coded as ordinal 0, 1, 2 variables) with the residuals as the outcome; and (4) Record the t-test statistic and corresponding p-value within each model for the test that the coefficient of the SNP in the linear regression was equal to 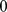. For the PennCAC data, SNP level 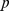-values were generated within Caucasians according to this same algorithm. For GLGC data, reported meta-analysis 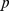-values that were generated in the same manner and then meta-analyzed were used in analysis. Transformations of these 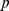-values and subsequent application of MixMAP proceeded as described above.

For all simulation studies, random gene level effects, 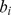 for 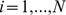, are simulated according to a two-component Gaussian mixture distribution with 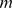 elements arising from a 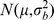 and the remaining 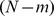 elements arising from a 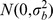 distribution, where 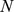 is the total number of genes under study and 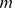 is the number of informative genes. SNP level z-scores are then generated according to the model 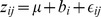 where 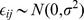. To begin for all simulations, the numbers of SNPs within each gene are set equal to the observed counts for the 31825 SNPs within the 2960 genes in the GLGC and PennCAC IBC subset. The median number of SNPs per gene is 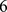, the mean is 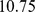 and the range is 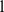 to 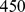. The first and third quartiles are equal to 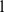 and 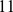, respectively. Notably, due to the LD structure within genes, we expect 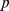-values to be correlated within these regions even under the complete null of no association between all genes and the trait under study. As a result, the gene level random effects, given by 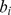 in the model above, are not identically equal to 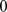 under this null. That is, even uninformative genes, whose latent effects are assumed to arise from a mean 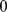 distribution, will have corresponding non-zero effects.

## Supporting Information

Supporting Information S1 **Clinical Studies.**
(PDF)

Supporting Information S2 **Summary of suggested novel genes/loci identified in GLGC data by MixMAP.**
(PDF)

Table S1 **Empirical Bayes (EB) estimates and corresponding prediction intervals for MixMAP supported genes in GLGC.** The Empirical Bayes estimate and corresponding prediction variance for each gene are used in the construction of an associated one-sided prediction interval. ^1^The upper limit of this interval is reported using a Bonferroni corrected α = 0.05/(2960). An upper limit that is less than 0 implies the gene's random effect (on the inverse normal transformed ranked *p*-values) is significantly less than 0. Since negative transformed *p*-values that are large in absolute value correspond to small *p*-values on the original scale, this implies the gene as a whole has a significant effect on LDL-C. *Highlighted rows correspond to novel loci not identified using single SNP analysis.
(PDF)

Table S2 **Empirical Bayes (EB) estimates and corresponding prediction intervals for MixMAP supported genes in PennCAC.** The Empirical Bayes estimate and corresponding prediction variance for each gene are used in the construction of an associated one-sided prediction interval. ^1^The upper limit of this interval is reported using a Bonferroni corrected α = 0.05/(2944). An upper limit that is less than 0 implies the gene's random effect (on the inverse normal transformed ranked *p*-values) is significantly less than 0. Since negative transformed *p*-values that are large in absolute value correspond to small *p*-values on the original scale, this implies the gene as a whole has a significant effect on LDL-C. None of these loci were identified using single SNP analysis.
(PDF)
