# Supplementary material for: Mixed Modeling of Meta-Analysis P-Values (MixMAP) Suggests Multiple Novel Gene Loci for Low Density Lipoprotein Cholesterol
Source: PLoS One. 2013 Feb 6;8(2):e54812. doi: 10.1371/journal.pone.0054812 (PMC3566142; doi:10.1371/journal.pone.0054812)
Supplement: Supporting Information S1 — Clinical Studies. (PDF) [file pone.0054812.s001.pdf]

S1: Clinical Studies

The Global Lipids Gene Consortium (GLGC): The clinical studies included in the GLGC GWAS of LDL-C and other plasma lipids are described in detail in Teslovich et al. [1].

The Penn Coronary Artery Calcification (PennCAC): The PennCAC resource has been described previously [2–4]. Briefly, PennCAC included European-ancestry subjects recruited to 3 separate studies at the University of Pennsylvania: the SIRCA (Study of Inherited Risk of Coronary Atherosclerosis) ($n = 799$), the PDHS (Penn Diabetes Heart Study) ($n = 782$), and the PAMSyN (Philadelphia Area Metabolic Syndrome Network) ($n = 480$). The SIRCA is a cross-sectional study of risk factors for coronary calcium in a community-based sample of asymptomatic subjects aged 30 to 75 years who had a family history of premature coronary artery disease (CAD). Subjects were excluded if they reported evidence of clinical CAD on screening questionnaire, had a history of diabetes mellitus, or had a serum creatinine $>$ 3.0 mg/dl. The PDHS is an ongoing, cross-sectional community-based study of type-2 diabetic subjects without clinical CAD or chronic kidney disease. Subjects were aged 35 to 75 years; had a clinical diagnosis of type-2 diabetes (defined as fasting blood glucose $\geq$ 126 mg/dl, 2-h postprandial glucose 200 mg/dl, or use of oral hypoglycemic agents/insulin in a subject greater than age 40 yr). Subjects were excluded if they had evidence of clinical CAD, a diagnosis of type 1 diabetes (insulin use prior to age 35), or a serum creatinine $>$ 2.5 mg/dl. PAMSyN is a cross-sectional study of patients with one or more metabolic syndrome risk factors, as defined by the National Cholesterol Education Program (NCEP) Adult Treatment Panel (ATP) III. Subjects with known diabetes or clinical atherosclerotic CVD were excluded. The SIRCA, PDHS, and PAMSyN samples are all single center studies that used the same clinical research center (U.Penn), nursing staff, CT scanner, and research laboratories. Plasma lipids were measured enzymatically (Cobas Fara II; Roche Diagnostic Systems, Somervile, NJ) in lipoprotein fractions after ultracentrifugation (β-quantification technique) in PDHS and in whole serum in SIRCA in a Penn's Center for Disease Controlcertified lipid laboratory. Analyses use LDL-C calculated by the Friedewald formula. For all human studies described, the University of Pennsylvania Institutional Review Board approved each study, and written informed consent was provided by all participants.

References

[1] Teslovich TM, Musunuru K, Smith AV, Edmondson AC, Stylianou IM, et al. (2010) Biological, clinical and population relevance of 95 loci for blood lipids. Nature 466: 707–713.

[2] Ferguson JF, Hinkle CC, Mehta NN, Bagheri R, Derohannessian SL, et al. (2012) Translational studies of lipoprotein-associated phospholipase a(2) in inflammation and atherosclerosis. J Am Coll Cardiol 59: 764–772.

[3] Shah R, Hinkle CC, Ferguson JF, Mehta NN, Li M, et al. (2011) Fractalkine is a novel human adipochemokine associated with type 2 diabetes. Diabetes 60: 1512–1518.

[4] Edmondson AC, Braund PS, Stylianou IM, Khera AV, Nelson CP, et al. (2011) Dense genotyping of candidate gene loci identifies variants associated with high-density lipoprotein cholesterol. Circ Cardiovasc Genet 4: 145–155.
