# Supplementary material for: Mixed Modeling of Meta-Analysis P-Values (MixMAP) Suggests Multiple Novel Gene Loci for Low Density Lipoprotein Cholesterol
Source: PLoS One. 2013 Feb 6;8(2):e54812. doi: 10.1371/journal.pone.0054812 (PMC3566142; doi:10.1371/journal.pone.0054812)
Supplement: Supporting Information S2 — Summary of suggested novel genes/loci identified in GLGC data by MixMAP. (PDF) [file pone.0054812.s002.pdf]

S2: Summary of suggested novel genes/loci identified in GLGC data by MixMAP

PKN2 (protein kinase N2): Chromosome 1p22.2 PKN2 is a serine/threonine kinase involved in small G-protein cell signaling. Conflicting data exist regarding an association with type 2 diabetes. There are no published data supporting a role in lipid or lipoprotein biology. TCEB1P19 and GTF2B are other genes in this region.

FN1 (fibronectin 1): Chromosome 2q34 FN1 encodes fibronectin, a glycoprotein present in a soluble dimeric form in plasma, and in a dimeric or multimeric form at the cell surface and in extracellular matrix. Fibronectin has multiple splice variants and is involved in cell adhesion and migration processes including embryogenesis, wound healing, blood coagulation, host defense, and metastasis. Fn1has been implicated in atherosclerosis in rodent models [1, 2] and is proposed to modulate lipoprotein uptake and retention in sub-endothelium. However, there are no published data supporting a role in plasma lipid or lipoprotein metabolism. DNAPTP3, AT1C, LOC646324, MREG, and ABCA12 are genes in this region.

UGT1A1 (UDP glucuronosyltransferase 1 family, polypeptide A1): Chromosome 2q37 UDP-glucuronosyltransferase, an enzyme of the glucuronidation pathway that transforms small lipophilic molecules, such as steroids, bilirubin, hormones, and drugs, into water-soluble, excretable metabolites. This gene is part of a complex locus that encodes several UDP-glucuronosyltransferases. The preferred substrate of this enzyme is bilirubin, although it also has moderate activity with simple phenols, flavones, and C18 steroids. Mutations in this gene result in Crigler-Najjar syndromes types I and II and in Gilbert syndrome. UGT1A1 is involved in drug and lipid metabolism. It has been implicated in gallstone formation plasma lipid levels in human as well as modulating the pharmaco-kinetics of lipid lowering therapies including statins and ezetimibe [3–6]. UGT1A1 therefore is a plausible candidate gene for LDL-C. A large family of UGT genes as well as DNAJB3 and HEART7B1 are in this region.

PPARG (Peroxisome proliferator-activated receptor-gamma 1): Chromosome 3p25 PPARG encodes a member of the PPAR subfamily of nuclear receptors. PPARs form heterodimers with retinoid X receptors (RXRs) and these heterodimers regulate transcription of various genes. The protein encoded by this gene is PPAR-gamma and is a regulator of adipocyte differentiation. PPARG is a required nuclear

hormone receptor for adipose differentiation and function, is implicated in Mendelian forms of lipodystrophy and insulin resistance as well as associated with insulin resistance, adiposity and type-2 diabetes in population studies. Although more literature supports its association with elevated triglycerides and low HDL-C (metabolic dyslipidemia) through its regulation of adipose and insulin resistance, a number of studies suggest a relationship with total cholesterol and LDL-C [7–10]. GSTM5P1, TIMP4, SYN2, LOC100505557, and TSEN are genes in this region.

DMGDH (dimethylglycine dehydrogenase): Chromosome 5q14.1 Dimethylglycine dehydrogenase (DMGDH) is a mitochondrial matrix enzyme involved in the metabolism of choline, converting dimethylglycine to sarcosine. Mutations in DMGDH have been described as a new inborn error of metabolism in human, DMGDH deficiency, associated with myopathy and fish like body odor [11]. There is no direct evidence for a role in lipid or LDL lipoprotein metabolism. BHMT genes, ARSB and LOC100505796 are other genes in this region

PPARD (peroxisome proliferator-activated receptor delta): Chromosome 6p21.2 PPARD encodes a member of the PPAR family. This protein is a potent inhibitor of ligand-induced transcription activity of PPAR alpha and PPAR gamma. It may function as an integrator of transcription repression and nuclear receptor signaling. Knockout studies in mice suggest the role of this protein in lipid metabolism, adipogeneis, energy utilization and type-2 diabetes. Several human genetic studies and early clinical trials support a role for PPARD in lipoprotein metabolism [12–16]. DEF6, MKRN6P, FANCE, and LOC100507672 are genes in this region.

CDK6 (cyclin-dependent kinase 6): Chromosome 7q21-q22 CDK6 encodes a member of the cyclin-dependent protein kinase (CDK) family. CDK family members are known to be important regulators of cell cycle progression. This kinase is a catalytic subunit of the protein kinase complex that is important for cell cycle G1 phase progression and G1/S transition. There are no published data implicating CDK6 in lipid or lipoprotein metabolism. FAM133B and LOC442710 are genes in this region.

VPS13B (vacuolar protein sorting 13 homolog B): Chromosome 8q22.2 VPS13B encodes a potential transmembrane protein that may function in vesicle-mediated transport and sorting of proteins within the cell. This protein may play a role in the development and the function of the eye, hematological

system, and central nervous system. Mutations in this gene have been associated with Cohen syndrome, an autosomal recessive disorder with variable developmental delay, visual disability, facial dysmorphisms and intermittent neutropenia. There are no published data supporting a role in lipid or lipoprotein biology but truncal obesity has been described in most patients with Cohen syndrome [17]. MIR599 and MIR875 are located within VPS13B.

GAD2 (glutamate decarboxylase 2): Chromosome 10p11.23 GAD2 encodes one of several forms of glutamic acid decarboxylase, identified as a major autoantigen in insulin-dependent diabetes. The enzyme encoded is responsible for catalyzing the production of gamma-aminobutyric acid from L-glutamic acid. A pathogenic role for this enzyme has been identified in the human pancreas since it has been identified as an autoantibody and an autoreactive T cell target in insulin-dependent diabetes. There are no published data supporting a role in lipid or lipoprotein biology. MYO3A and APBB1IP are genes in this region

GAB2 (GRB2-associated binding protein 2): Chromosome 11q14.1 GAB2 is a member of the GRB2-associated binding protein (GAB) gene family. These proteins contain pleckstrin homology (PH) domain, and bind SHP2 tyrosine phosphatase and GRB2 adapter protein. They act as adapters for transmitting various signals in response to stimuli through cytokine and growth factor receptors, and T- and B-cell antigen receptors. The protein encoded by this gene is the principal activator of phosphatidylinositol-3 kinase in response to activation of the high affinity IgE receptor. NARS2, USP35, KCTD21, ZNF75CP and LOC100506220 are genes in this region.

APOH (Apolipoprotein H): Chromosome 17q23-qter Apolipoprotein H, also named beta-2 glycoprotein I, has been implicated in a variety of physiologic pathways including lipoprotein metabolism, coagulation, and the production of antiphospholipid autoantibodies. APOH may be a required cofactor for anionic phospholipid binding by the antiphospholipid autoantibodies found in sera of many patients with lupus and primary antiphospholipid syndrome. APOH is found on several classes of lipoproteins and is involved in the activation of lipoprotein lipase in lipid metabolism. There are several human genetic studies suggesting a role for APOH in cholesterol and triglyceride metabolism and in the association with plasma lipoproteins across multiple ethnic groups [18–21]. PRKCA and PSMD7P1 are genes in this region.

NPC1 (Niemann-Pick disease, type C1): Chromosome 18q11-q12 NPC1 encodes a large protein that resides in the limiting membrane of endosomes and lysosomes and mediates intracellular cholesterol trafficking via binding of cholesterol to its N-terminal domain This protein transports low-density lipoproteins to late endosomal/lysosomal compartments where they are hydrolized and released as free cholesterol. Defects in this gene cause Niemann-Pick type C disease, a rare autosomal recessive neurodegenerative disorder characterized by over accumulation of cholesterol and glycosphingolipids in late endosomal/lysosomal compartments [22–25]. RI0K3, C18orf8 and ANKRD29 are genes in this region.

References

[1] Duner P, To F, Beckmann K, Bjorkbacka H, Fredrikson GN, et al. (2011) Immunization of apoE-/- mice with aldehyde-modified fibronectin inhibits the development of atherosclerosis. Cardiovasc Res 91: 528–536.

[2] Dietrich T, Perlitz C, Licha K, Stawowy P, Atrott K, et al. (2007) ED-B fibronectin (ED-B) can be targeted using a novel single chain antibody conjugate and is associated with macrophage accumulation in atherosclerotic lesions. Basic Res Cardiol 102: 298–307.

[3] Oswald S, Haenisch S, Fricke C, Sudhop T, Remmler C, et al. (2006) Intestinal expression of P-glycoprotein (ABCB1), multidrug resistance associated protein 2 (ABCC2), and uridine diphosphate-glucuronosyltransferase 1A1 predicts the disposition and modulates the effects of the cholesterol absorption inhibitor ezetimibe in humans. Clin Pharmacol Ther 79: 206–217.

[4] Smiderle L, Galvao AC, Fontana C, Wender MC, Agnes G, et al. (2011) Evaluation of UGT1A1 and SULT1A1 polymorphisms with lipid levels in women with different hormonal status. Gynecol Endocrinol 27: 20–26.

[5] Bae JW, Choi CI, Lee JH, Jang CG, Chung MW, et al. (2011) Effects of UDP-glucuronosyltransferase polymorphisms on the pharmacokinetics of ezetimibe in healthy subjects. Eur J Clin Pharmacol 67: 39–45.

[6] Osabe M, Sugatani J, Fukuyama T, Ikushiro S, Ikari A, et al. (2008) Expression of hepatic UDP-glucuronosyltransferase 1A1 and 1A6 correlated with increased expression of the nuclear constitutive

androstane receptor and peroxisome proliferator-activated receptor alpha in male rats fed a high-fat and high-sucrose diet. Drug Metab Dispos 36: 294–302.

[7] Fruchart JC, Duriez P, Staels B (1999) Peroxisome proliferator-activated receptor-alpha activators regulate genes governing lipoprotein metabolism, vascular inflammation and atherosclerosis. Curr Opin Lipidol 10: 245–257.

[8] Meirhaeghe A, Tanck MW, Fajas L, Janot C, Helbecque N, et al. (2005) Study of a new PPARgamma2 promoter polymorphism and haplotype analysis in a French population. Mol Genet Metab 85: 140–148.

[9] Johansson LE, Danielsson P, Norgren S, Marcus C, Ridderstrale M (2009) Interaction between PPARG Pro12Ala and ADIPOQ G276T concerning cholesterol levels in childhood obesity. Int J Pediatr Obes 4: 119–125.

[10] Garg A (2011) Clinical review: Lipodystrophies: genetic and acquired body fat disorders. J Clin Endocrinol Metab 96: 3313–3325.

[11] Binzak BA, Wevers RA, Moolenaar SH, Lee YM, Hwu WL, et al. (2001) Cloning of dimethylglycine dehydrogenase and a new human inborn error of metabolism, dimethylglycine dehydrogenase deficiency. Am J Hum Genet 68: 839–847.

[12] Skogsberg J, Kannisto K, Cassel TN, Hamsten A, Eriksson P, et al. (2003) Evidence that peroxisome proliferator-activated receptor delta influences cholesterol metabolism in men. Arterioscler Thromb Vasc Biol 23: 637–643.

[13] Skogsberg J, McMahon AD, Karpe F, Hamsten A, Packard CJ, et al. (2003) Peroxisome proliferator activated receptor delta genotype in relation to cardiovascular risk factors and risk of coronary heart disease in hypercholesterolaemic men. J Intern Med 254: 597–604.

[14] Hu C, Jia W, Fang Q, Zhang R, Wang C, et al. (2006) Peroxisome proliferator-activated receptor (PPAR) delta genetic polymorphism and its association with insulin resistance index and fasting plasma glucose concentrations in Chinese subjects. Diabet Med 23: 1307–1312.

[15] Burch LR, Donnelly LA, Doney AS, Brady J, Tommasi AM, et al. (2010) Peroxisome proliferator-activated receptor-delta genotype influences metabolic phenotype and may influence lipid response to

statin therapy in humans: a genetics of diabetes audit and research Tayside study. J Clin Endocrinol Metab 95: 1830–1837.

[16] Sanderson LM, Degenhardt T, Koppen A, Kalkhoven E, Desvergne B, et al. (2009) Peroxisome proliferator-activated receptor beta/delta (PPARbeta/delta) but not PPARalpha serves as a plasma free fatty acid sensor in liver. Mol Cell Biol 29: 6257–6267.

[17] Katzaki E, Pescucci C, Uliana V, Papa FT, Ariani F, et al. (2007) Clinical and molecular characterization of Italian patients affected by Cohen syndrome. J Hum Genet 52: 1011–1017.

[18] Kamboh MI, Bunker CH, Aston CE, Nestlerode CS, McAllister AE, et al. (1999) Genetic association of five apolipoprotein polymorphisms with serum lipoprotein-lipid levels in African blacks. Genet Epidemiol 16: 205–222.

[19] Tsunoda K, Harihara S, Dashnyam B, Semjidmaa D, Yamaguchi Y, et al. (2002) Apolipoprotein E and H polymorphisms in Mongolian Buryat: allele frequencies and relationship with plasma lipid levels. Hum Biol 74: 659–671.

[20] Kamboh MI, Sanghera DK, Mehdi H, Nestlerode CS, Chen Q, et al. (2004) Single nucleotide polymorphisms in the coding region of the apolipoprotein H (beta2-glycoprotein I) gene and their correlation with the protein polymorphism, anti-beta2glycoprotein I antibodies and cardiolipin binding: description of novel haplotypes and their evolution. Ann Hum Genet 68: 285–299.

[21] Leduc MS, Shimmin LC, Klos KL, Hanis C, Boerwinkle E, et al. (2008) Comprehensive evaluation of apolipoprotein H gene (APOH) variation identifies novel associations with measures of lipid metabolism in GENOA. J Lipid Res 49: 2648–2656.

[22] Schroeder F, Gallegos AM, Atshaves BP, Storey SM, McIntosh AL, et al. (2001) Recent advances in membrane microdomains: rafts, caveolae, and intracellular cholesterol trafficking. Exp Biol Med (Maywood) 226: 873–890.

[23] Scott C, Ioannou YA (2004) The NPC1 protein: structure implies function. Biochim Biophys Acta 1685: 8–13.

[24] Chang TY, Chang CC, Ohgami N, Yamauchi Y (2006) Cholesterol sensing, trafficking, and esterification. Annu Rev Cell Dev Biol 22: 129–157.

[25] Dierks T, Schlotawa L, Frese MA, Radhakrishnan K, von Figura K, et al. (2009) Molecular basis of multiple sulfatase deficiency, mucolipidosis II/III and Niemann-Pick C1 disease - Lysosomal storage disorders caused by defects of non-lysosomal proteins. Biochim Biophys Acta 1793: 710–725.
