# Supplementary material for: Mixed Modeling of Meta-Analysis P-Values (MixMAP) Suggests Multiple Novel Gene Loci for Low Density Lipoprotein Cholesterol
Source: PLoS One. 2013 Feb 6;8(2):e54812. doi: 10.1371/journal.pone.0054812 (PMC3566142; doi:10.1371/journal.pone.0054812)
Supplement: Table S1 — Empirical Bayes (EB) estimates and corresponding prediction intervals for MixMAP supported genes in GLGC. The Empirical Bayes estimate and corresponding prediction variance for each gene are used in the construction of an associated one-sided prediction interval. 1The upper limit of this interval is reported using a Bonferroni corrected α = 0.05/(2960). An upper limit that is less than 0 implies the gene's random effect (on the inverse normal transformed ranked p-values) is significantly less than 0. Since negative transformed p-values that are large in absolute value correspond to small p-values on the original scale, this implies the gene as a whole has a significant effect on LDL-C. *Highlighted rows correspond to novel loci not identified using single SNP analysis. (PDF) [file pone.0054812.s003.pdf]

Ch #	Gene Name	EB Estimate	Prediction Variance	PI Upper Limit[1]	# of SNPs
1	PCSK9	-1.506	0.022	-0.896	34
*1	PKN2	-0.88	0.033	-0.127	21
1	CELSR2	-2.399	0.031	-1.674	23
1	PSRC1	-1.557	0.077	-0.41	7
1	MYBPHL	-1.769	0.06	-0.756	10
1	SORT1	-1.981	0.026	-1.317	28
2	APOB	-1.969	0.015	-1.454	49
*2	FN1	-0.674	0.017	-0.132	44
*2	UGT1A1	-0.912	0.034	-0.143	20
2	ABCG8	-1.207	0.033	-0.454	21
2	ABCG5	-1.191	0.028	-0.493	25
2	LCT	-1.169	0.056	-0.192	11
3	PPARG	-0.801	0.015	-0.285	49
5	HMGCR	-1.451	0.056	-0.474	11
5	COL4A3BP	-1.54	0.064	-0.488	9
*5	DMGDH	-0.903	0.036	-0.117	19
5	SGCD	-0.663	0.012	-0.215	66
5	HAVCR2	-1.006	0.056	-0.029	11
6	HLA-DRA	-0.95	0.049	-0.033	13
6	MICA	-1.159	0.034	-0.39	20
6	LTA	-0.855	0.038	-0.051	18
6	C2	-1.045	0.046	-0.154	14
6	TAP2	-0.762	0.028	-0.063	25
*6	PPARD	-0.609	0.017	-0.073	45
6	LPAL2	-1.091	0.064	-0.039	9
6	LPA	-1.212	0.025	-0.558	29
7	NPC1L1	-1.311	0.046	-0.421	14
*7	CDK6	-0.779	0.017	-0.231	43
*8	VPS13B	-0.757	0.024	-0.112	30
8	TRIB1	-1.672	0.015	-1.157	49
9	ABO	-1.739	0.044	-0.873	15
9	ABCA1	-0.532	0.007	-0.197	121
*10	GAD2	-0.94	0.032	-0.201	22
11	FADS1	-1.836	0.064	-0.784	9
*11	GAB2	-1.101	0.049	-0.184	13
11	BUD13	-1.366	0.041	-0.522	16
11	ZNF259	-1.423	0.064	-0.371	9
11	APOA5	-1.359	0.084	-0.154	6
12	ALDH2	-1.187	0.077	-0.04	7
12	TCF1	-1.134	0.049	-0.217	13
16	CETP	-1.339	0.017	-0.798	44
*17	APOH	-0.77	0.028	-0.071	25

Continued on next page

Table 1 – continued from previous page – Lipids GWAS Meta-analysis data

Ch #	Gene Name	EB Estimate	Prediction Variance	PI Upper Limit[1]	# of SNPs
*18	NPC1	-0.917	0.041	-0.073	16
19	LDLR	-2.021	0.026	-1.356	28
19	NCAN	-0.985	0.052	-0.039	12
19	BCL3	-1.495	0.084	-0.29	6
19	PVRL2	-1.811	0.041	-0.967	16
19	APOE	-1.236	0.07	-0.139	8
