# Supplementary material for: Mixed Modeling of Meta-Analysis P-Values (MixMAP) Suggests Multiple Novel Gene Loci for Low Density Lipoprotein Cholesterol
Source: PLoS One. 2013 Feb 6;8(2):e54812. doi: 10.1371/journal.pone.0054812 (PMC3566142; doi:10.1371/journal.pone.0054812)
Supplement: Table S2 — Empirical Bayes (EB) estimates and corresponding prediction intervals for MixMAP supported genes in PennCAC. The Empirical Bayes estimate and corresponding prediction variance for each gene are used in the construction of an associated one-sided prediction interval. 1The upper limit of this interval is reported using a Bonferroni corrected α = 0.05/(2944). An upper limit that is less than 0 implies the gene's random effect (on the inverse normal transformed ranked p-values) is significantly less than 0. Since negative transformed p-values that are large in absolute value correspond to small p-values on the original scale, this implies the gene as a whole has a significant effect on LDL-C. None of these loci were identified using single SNP analysis. (PDF) [file pone.0054812.s004.pdf]

Ch #	Gene Name	EB Estimate	Prediction Variance	PI Upper Limit[1]	# of SNPs
1	CELSR2	-1.083	0.029	-0.375	23
2	IL1R2	-0.68	0.02	-0.093	37
4	FGF2	-0.88	0.027	-0.194	25
4	TNIP3	-0.95	0.041	-0.112	14
6	LPA	-0.672	0.025	-0.015	28
7	GRM3	-1.297	0.023	-0.674	32
8	VPS13B	-0.653	0.024	-0.014	30
